# Elucidating the Mechanism of *Trypanosoma cruzi* Acquisition by Triatomine Insects: Evidence from a Large Field Survey of *Triatoma infestans*

**DOI:** 10.3390/tropicalmed5020087

**Published:** 2020-06-01

**Authors:** Aaron W. Tustin, Ricardo Castillo-Neyra, Laura D. Tamayo, Renzo Salazar, Katty Borini-Mayorí, Michael Z. Levy

**Affiliations:** 1 Department of Environmental Health Sciences, Johns Hopkins Bloomberg School of Public Health, Baltimore, MD 21205, USA; aarontustin2@gmail.com; 2 Zoonotic Disease Research Lab, One Health Unit, School of Public Health and Administration, Universidad Peruana Cayetano Heredia, Lima, Lima Province 15102, Peru; cricardo@upenn.edu (R.C.-N.); laura.tamayo@upch.pe (L.D.T.); rendaths@gmail.com (R.S.); yttakbm@gmail.com (K.B.-M.); 3 Department of Biostatistics, Epidemiology & Informatics, Perelman School of Medicine of the University of Pennsylvania, Philadelphia, PA 19104, USA

**Keywords:** *Trypanosoma cruzi*, *Triatoma infestans*, Chagas disease, parasite prevalence, coprophagy

## Abstract

Blood-sucking triatomine bugs transmit the protozoan parasite *Trypanosoma cruzi*, the etiologic agent of Chagas disease. We measured the prevalence of *T. cruzi* infection in 58,519 *Triatoma infestans* captured in residences in and near Arequipa, Peru. Among bugs from infected colonies, *T. cruzi* prevalence increased with stage from 12% in second instars to 36% in adults. Regression models demonstrated that the probability of parasite acquisition was roughly the same for each developmental stage. Prevalence increased by 5.9% with each additional stage. We postulate that the probability of acquiring the parasite may be related to the number of feeding events. Transmission of the parasite does not appear to be correlated with the amount of blood ingested during feeding. Similarly, other hypothesized transmission routes such as coprophagy fail to explain the observed pattern of prevalence. Our results could have implications for the feasibility of late-acting control strategies that preferentially kill older insects.

## 1. Introduction

*Trypanosoma cruzi*, the protozoan parasite that causes Chagas disease, is transmitted to humans via the feces of blood-sucking triatomine insects (Hemiptera: Reduviidae) such as *Triatoma infestans*, the major vector of Chagas disease in much of South America [1]. Triatomines pass through five nymphal instar stages before becoming adults. All triatomine stages are at risk for acquiring *T. cruzi* because they all ingest blood meals. Insects may also become infected when they engage in behaviors such as coprophagy [2]. Once acquired, infection with *T. cruzi* is persistent [3]. Therefore, in well-stablished colonies with stable transmission patterns, *T. cruzi* prevalence is expected to increase monotonically with the insects’ developmental stage. The general shape (e.g., linear, exponential, logarithmic) of the rising prevalence curve may give clues as to the method of transmission. With respect to Chagas disease control, it may be important to ascertain the shape of this relationship between *T. cruzi* prevalence and stage.

**Figure 1:**
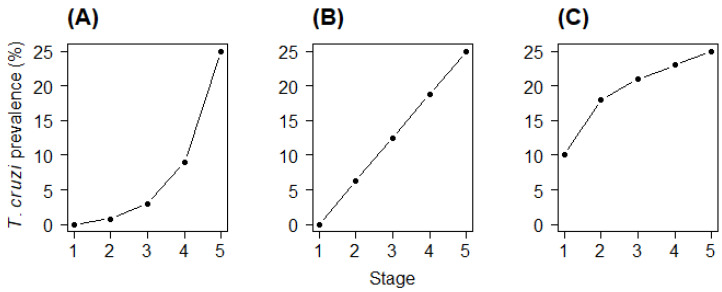
Hypothetical shapes of the relationship between triatomine stage and prevalence of infection with *Trypanosoma cruzi* under three hypotheses. (**A**) Acquisition of the parasite depends upon the quantity of blood ingested (Blood Hypothesis); (**B**) Parasite acquisition depends upon the number of exposure opportunities (Bites Hypothesis); (**C**) Early instar nymphs frequently acquire the parasite via coprophagy, while older instars acquire the parasite at a lower rate via ingestion of blood from mammalian hosts (Coprophagy Hypothesis). Note: the scale of the vertical axis is for illustrative purpose only and does not correspond to actual data; rather, panels (**A**) through (**C**) demonstrate three different theoretical shapes that could each produce 25% prevalence in fifth instars.

In this report we show the stage-prevalence of *T. cruzi* in a large sample of *T. infestans* captured during vector control and surveillance activities in Arequipa, Peru. These data allow us to test three hypotheses of *T. cruzi* acquisition by triatomines. Our first hypothesis, hereafter called the Blood Hypothesis, is that the probability of infection with *T. cruzi* depends upon the amount of blood ingested. Observations of laboratory-reared insects indicate that the quantity of blood ingested by *T. infestans* increases rapidly and nonlinearly with stage [4] Thus, if the Blood Hypothesis is correct, there might be a rapid nonlinear rise in *T. cruzi* prevalence with stage (Figure 1A). A second possibility is that an insect’s risk of infection depends upon the number of opportunities to acquire the parasite (Bites Hypothesis), rather than the amount of blood ingested. Under this hypothesis, *T. cruzi* prevalence among instars might rise in a roughly linear fashion with stage (Figure 1B), as all nymphs probably take roughly equal numbers of bites. 

The third hypothesis is that triatomines frequently acquire *T. cruzi* via coprophagy (Coprophagy Hypothesis). Triatomines ingest small amounts of feces of older insects in order to acquire bacterial symbionts necessary for digestion of blood meals [5]. Presumably, it is the early instars that ingest feces, as nymphs of some species fail to mature in the absence of symbionts [6]. An isolated experiment has suggested that *T. infestans* may also acquire *T. cruzi* by this route [2]. If parasite transmission via coprophagy is common under field conditions, then *T. cruzi* prevalence may increase relatively quickly among first and second instars, followed by a less rapid increase in older nymphs that acquire the parasite only via blood feeding (Figure 1C).

## 2. Materials and Methods

Between 2008 and 2014, workers from the Arequipa Ministry of Health and the Universidad Peruana Cayetano Heredia/University of Pennsylvania (UPCH/Penn) Zoonotic Disease Research Laboratory collected triatomines from urban and peri-urban households in and around the city of Arequipa. Located at 2300 m above sea level in an arid zone, Arequipa is the second largest city in Perú, home to 1,008,290 people [7]. Since the 1960s, the population has been growing rapidly due to an influx of immigrants, most of whom have settled on hillsides in the periphery of the city. These settlements are characterized by poorly built housing, mostly constructed with volcanic stone and bricks without mortar, where there is a greater presence of food animals such as guinea pigs or chickens [8]. On the other hand, closer to the center are the oldest neighborhoods (dating back to the late 19th and early 20th centuries), which are inhabited by people with a higher socioeconomic status [9]. The prevalence of *Triatoma infestans*-infested houses between these two types of zones are different: Households in hillside neighborhoods are more likely to be infested than are households in neighborhoods close to the center of the city [10]. In general, the distribution of *Triatoma infestans*, the only insect vector of *T. cruzi* in the city, occurs both by active dispersal, through flying and walking, where the streets are barriers, and by passive dispersal through human movement and migration [10,11,12].

The collection methods have been described previously [13,14]. All insects were categorized by sex (for adults), stage, and site. A site was defined as all rooms and peri-domestic areas, such as animal enclosures, associated with a single dwelling. First instar insects did not undergo further analysis. The remaining triatomines were inspected for *T. cruzi* at the UPCH/Penn Zoonotic Disease Research Center in Arequipa, using standard techniques [15]. Briefly, we extracted feces by applying pressure to each insect’s abdomen with forceps. Feces were diluted in 10 μL normal saline and examined microscopically at 400× magnification for the presence of parasites.

We built regression models to explore the relationship between *T. cruzi* prevalence and developmental stage. Due to uncertainty surrounding the total number and size of blood meals taken by adult insects, we excluded adults from our analysis. To avoid biasing our results by including a large number of insects with no potential exposure, we also excluded vectors captured at sites with no infected triatomines. 

Under the Bites Hypothesis, in which the risk of acquiring the parasite is the same for each stage, the stage-prevalence relationship is given by the following cumulative binomial probability distribution:

*P*(*S*) = 1 − (1 − *p_bite_*)*^S^* (1)

where *P* is the observed infection prevalence in nymphs of stage *S* (i.e., 1 ≤ *S* ≤ 5), and *p_bite_* is the probability of acquiring the parasite during any given stage (Note that when *S* ≤ 5 and *p* is sufficiently small, Equation (1) can be approximated by the linear function *P*(*S*) ≈ *pS*. For example, if *p* = 0.01 and *S* = 5, then *P*(*S*) = 0.049 and *pS* = 0.05. This is why we hypothesized that the stage-prevalence relationship might appear roughly linear if the Bites Hypothesis were correct). To test the Bites Hypothesis, we used nonlinear least squares regression to fit Equation (1) to the observed data. 

To test the Blood Hypothesis, we assumed that the risk of acquiring the parasite is the same for each milligram of blood ingested. If *B*(*S*) represents the cumulative quantity (mg) of blood ingested by the average nymph of stage *S*, then the stage-prevalence relationship under the Blood Hypothesis is given by:

*P*(*S*) = 1 − (1 − *p_blood_*)*^B^*^(*S*)^ (2)

where *p_blood_* is the probability of acquiring the parasite after ingesting 1 mg of blood. The primary difference between Equations (1) and (2) is that the exponent of Equation (1) increases linearly with stage, whereas the exponent of Equation (2) increases nonlinearly. We estimated *B*(*S*) from *T. infestans* feeding data in a prior study [4]. Our estimates were *B*(1) = 7.1 mg, *B*(2) = 25.4 mg, *B*(3) = 87.8 mg, *B*(4) = 302.8 mg, and *B*(5) = 1072.8.0 mg (see Appendix A for derivation). We then used nonlinear least squares regression, with *B*(*S*) fixed at these values, to fit Equation (2) and estimate *p_blood_* given the observed data [4]. 

Because the mathematical relationship between feces ingestion and infection transmission is unknown, we did not create a specific model to test the Coprophagy Hypothesis. Instead, we made a qualitative assessment of the importance of feces-mediated transmission by examining whether the other two models underestimated the infection prevalence in early-stage insects. 

We used the Akaike information criterion (AIC) [16] to guide model selection. Analyses were performed in R version 3.6.3 (R Foundation for Statistical Computing, Vienna, Austria).

## 3. Results

**Figure 2:**
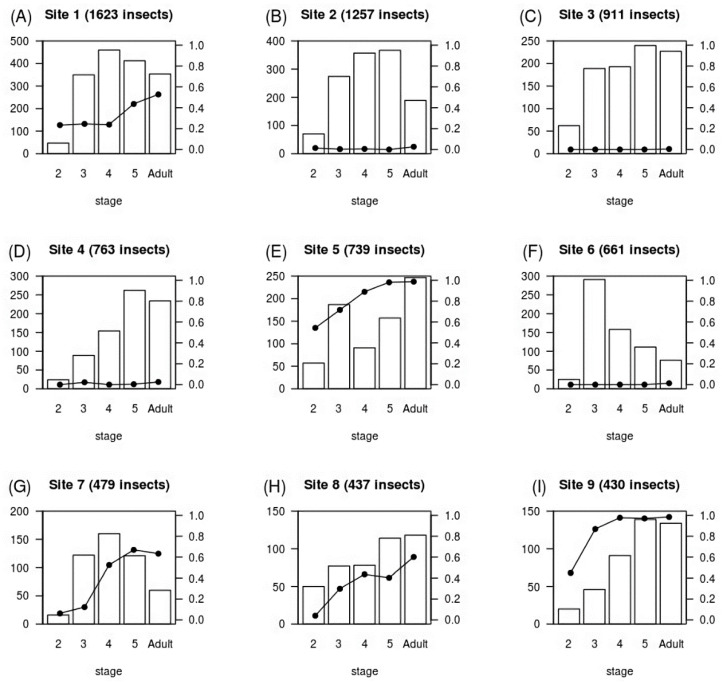
Distribution of developmental stage and *Trypanosoma cruzi* infection status in *Triatoma infestans* from nine households in Arequipa, Peru. Shown are the nine infected sites with the largest number of captured insects. White and black bars and left vertical axis labels: total number of insects. Black circles and right vertical axis labels: fraction of insects with *T. cruzi*.

**Figure 3:**
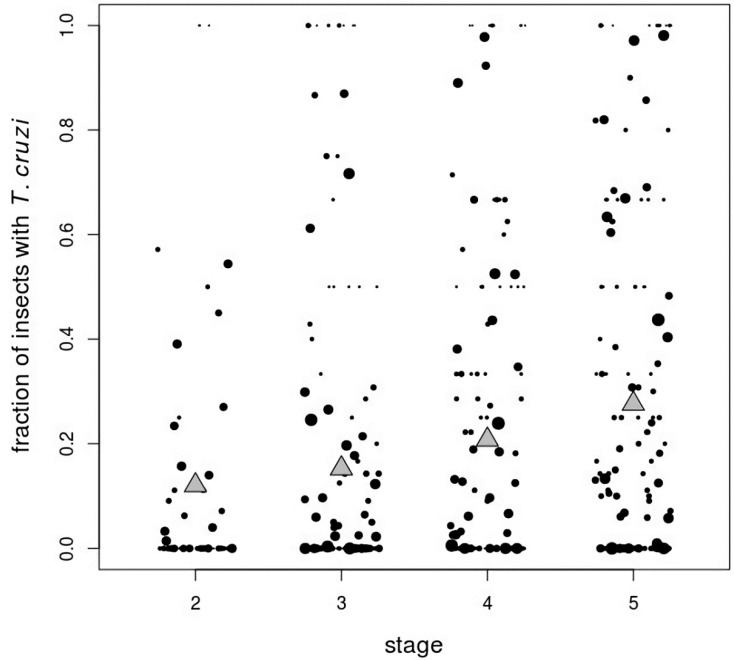
Fraction of nymph *Triatoma infestans* infected with *Trypanosoma cruzi* at 188 sites with at least one infected insect. Black circles represent individual sites; sizes of circles are proportional to the logarithm of the number of insects captured. The bottom edges of the gray triangles show the mean fraction of insects with *T. cruzi* across all infected sites.

We captured and analyzed 58,519 triatomines from 4138 sites. There were 188 sites (4.5%), harboring 15,252 insects, with at least one *T. cruzi*-infected triatomine. The stage distribution and *T. cruzi* prevalence of captured insects varied considerably between sites, as can be seen in Figure 2, which shows the nine infected sites with the highest number of insects. Second instars were underrepresented at most sites, possibly because it is difficult to find and capture these small nymphs. The population structure of third instars through adults was flat in some colonies (e.g., Figure 2A,C), while other colonies were skewed toward younger (Figure 2F) or older (Figure 2D) insects. These differences may be due to the age of the colonies, or they may represent heterogeneity, across sites, in stage-dependent survival. *T. cruzi* prevalence was high in some colonies (e.g., Figure 2E,I), while other colonies had only one or a few infected insects (e.g., Figure 2B–D). We speculate that the colonies with very few infected insects may represent sites where *T. cruzi* was recently introduced, perhaps via a newly infected host or migration of an infected triatomine from a nearby household. Averaged across all 188 sites, the mean prevalence of *T. cruzi* infection rose monotonically from 12% in second instars to 36% in adults (Table 1 and Figure 3). 

**Figure 4:**
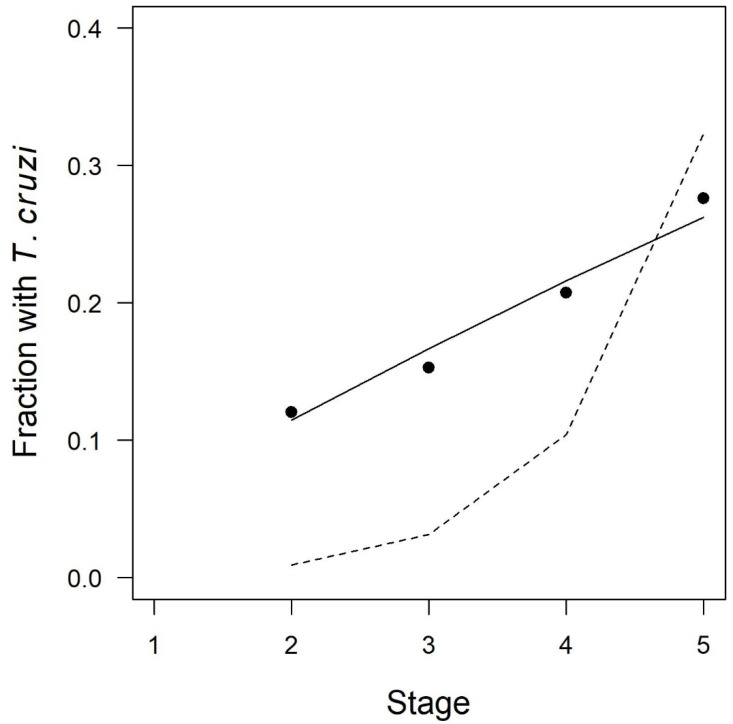
Fraction of triatomines infected with *Trypanosoma cruzi* as a function of stage. Filled circles: mean values observed in 15,252 insects captured at 188 infected sites. Dashed line: Best-fit regression model under the Blood Hypothesis that assumed an equal probability of infection with each milligram of blood ingested (*Pr(infection*) = 1 − (1 − 0.00036)^*Nblood,* where *Nblood* is the number of mg of blood ingested). Solid line: Best-fit regression model under the Bites Hypothesis that assumed an equal probability of infection during any given stage (*Pr(infection*) = 1 − (1 − 0.059)^*N*).

Regression modeling demonstrated that the Bites Hypothesis was a better fit to the observations of mean *T. cruzi* prevalence versus stage (Table 2 and Figure 4). In the Bites Hypothesis model, the best-fit parameter was *p_bite_* = 0.059, indicating a 5.9% probability of acquiring the parasite during any given stage. Visual inspection revealed that the Bites Hypothesis model fit the data well (Figure 4), and the model had an AIC of 11,672. In contrast, the Blood Hypothesis regression model was a very poor visual fit with a significantly higher (i.e., worse) AIC of 12,302. The observations of mean prevalence versus stage did not exhibit the excess prevalence in early nymphs that would occur if coprophagy were a primary driver of infection.

## 4. Discussion

In a large field survey of *Triatoma infestans* captured in Arequipa, Peru, we demonstrate that the probability of *Trypanosoma cruzi* acquisition is the same at each developmental stage. This result suggests that acquisition of *T. cruzi* depends on the number of feeding opportunities (i.e., bites) and not on the quantity of blood ingested. A possible explanation for this finding is that newly infected mammalian hosts may undergo a rapid logistic increase in the number of circulating parasites. In other words, hosts may go from no parasitemia to a high level of infectiousness very quickly [17,18]. Vectors that feed on such hosts may be almost certain to acquire the parasite, regardless of the size of the blood meal they ingest. Hosts may spend only a brief amount of time at intermediate levels of parasitemia, during which time vectors acquire the parasite in proportion to the amount of blood ingested. Although this interpretation assumes that instar stage is proportional to the number of blood meals, we note that the exact relationship between these two quantities is uncertain. Some nymphs may take only one blood meal per molt, while others may take multiple blood meals per developmental stage, particularly if their feeding is interrupted before engorgement [19,20].

Our results are in general agreement with those of a prior study that reported increasing *T. cruzi* prevalence with stage in a smaller sample of wild-caught *T. infestans* nymphs and adults [21]. As in the previous study, a vector control program was ongoing in Arequipa during the time period when we collected insects. These control efforts reduced transmission of *T. cruzi* to humans [14] and decreased the population of vectors. We also confirm the results of a previous small study (n < 200) of laboratory-reared fifth instar triatomines used for xenodiagnosis, in which there was no correlation between blood meal size and the probability of *T. cruzi* infection [22].

Several reports have cast doubt on the importance of coprophagy as a route of *T. cruzi* acquisition [17,23,24]. The present study provides complementary epidemiologic evidence, from *T. infestans* captured in natural habitats, against the coprophagy route of transmission. However, since we were unable to examine first instars, more work is needed to quantify the precise role, if any, of coprophagy. It should also be noted that triatomines’ need for bacterial symbionts and coprophagy have not been established with certainty. Wild-caught triatomines harbor diverse gut bacteria [6], which suggests they can acquire symbionts from routes other than coprophagy, and many triatomine species (including *T. protracta*, *T. rubida*, and *Rhodnius prolixus*) develop normally when raised in sterile environments [25]. 

Limitations of this study include our inability to measure the infection prevalence among first instars; our averaging of the infection prevalence across all sites, which may have oversimplified a more complex pattern of parasite transmission such as may occur in a metapopulation; and uncertainty in our assumptions regarding the number and size of blood meals consumed by triatomines of different stages. Also, the number of infected insects may have been underestimated due to the method used to detect the presence of infection in this study, which has been reported to be less sensitive than other methods such as complete insect dissection or PCR. Another limitation is that migration of infected triatomines could have biased our prevalence estimates by causing us to include colonies without well-established stable patterns of infection versus stage.

The observed pattern of *T. cruzi* prevalence versus stage could have implications for Chagas disease control strategies. Insecticides and biological control methods that preferentially kill older insects have relatively less effect on an insect’s reproductive fitness and, in theory, are less likely to be rendered obsolete by evolution of resistance mechanisms [26]. Late-acting agents have been suggested as a means to combat infections including malaria [27] and Chagas disease [28]. Had we observed a highly linear increase in *T. cruzi* prevalence with stage, with few infected early-stage nymphs, we could have made a strong case for such late-acting methods. Instead, we found a substantial rate of *T. cruzi* infection in second and third instars. Although our results suggest that disproportionate killing of older triatomines may not be useful, these findings should be interpreted with caution. The probability that *T. cruzi* will be transmitted to humans depends upon several factors that we did not measure. Behavioral and physiological factors may make younger triatomines less likely to transmit the parasite. Younger instars may be less likely than adult insects to defecate on hosts [29]; may defecate longer after taking a blood meal [30]; and may be less likely to survive the parasite’s incubation period, since survival of infected triatomines appears to be affected by the ratio of ingested blood to body weight [31]. Further studies will be needed to determine the relative risk of feces-mediated transmission posed by younger and older triatomines, which in turn would inform the decision of whether to pursue late-acting control strategies.

## Figures and Tables

**Table 1:** *Trypanosoma cruzi* infection status by developmental stage, for 15,252 triatomines captured in 188 infected colonies in Arequipa, Peru.

Stage	Number Infected/Total Insects (%)
Second instar	125/1037 (12.1)
Third instar	512/3352 (15.3)
Fourth instar	687/3310 (20.8)
Fifth instar	1060/3838 (27.6)
Adult	1326/3715 (35.7)
Male	853/2225 (38.3)
Female	473/1490 (31.7)
Total	3710/15,252 (24.3)

**Table 2:** Results of regression modeling to test two hypotheses of *T. cruzi* transmission to *T. infestans*.

Model Name	Fitted Parameter	Best-Fit Parameter (95% Confidence Interval)	AIC
Bites Hypothesis	*p_bite_*: probability of parasite acquisition during any given stage	*p_bite_* = 0.059 (0.057 to 0.061)	11,672
Blood Hypothesis	*p_blood_*: probability of parasite acquisition with each milligram of blood ingested	*p_blood_* = 0.00036 (0.00035 to 0.00038)	112,302

Abbreviation: AIC, Akaike information criterion.

## Appendix A

Juarez [4] measured blood meal sizes of laboratory-reared *T. infestans* under a variety of conditions of feed source, ambient temperature, and *T. cruzi* infection status. Of the eight groups of insects studied by Juarez, the group that was uninfected, fed on chicken, and maintained at 25 °C (77 °F) was closest to the natural conditions encountered by wild insects in Arequipa. Under these conditions, Juarez found that the quantity of blood ingested during each stage increased from 7.1 mg in first instars to 770 mg in fifth instars. We used these data to estimate the cumulative quantity of blood, *B*(*S*), ingested by captured wild *T. infestans* nymphs (Table A1). We assumed that insects we captured just before a molt had already ingested the stage-specific quantity of blood reported by Juarez. Under this assumption, the mean cumulative blood ingested by captured insects is simply the sum of the blood ingested during the current and all prior stages.

**Table A1:** Blood meal sizes observations for laboratory reared insects [4] and estimates of cumulative blood ingested by captured wild *Triatoma infestans* nymphs.

Stage	Median Quantity of Blood Ingested during this Stage (mg)	Estimated Cumulative Blood Ingested during Lifetime Prior to Capture (mg)
First instar	7.1	7.1
Second instar	18.3	25.4
Third instar	62.4	87.8
Fourth instar	215.0	302.8
Fifth instar	770.0	1072.8
